# Protocol for the simultaneous isolation of DNA, RNA, and miRNA from a single archived Kaposi sarcoma biopsy

**DOI:** 10.1016/j.xpro.2024.103365

**Published:** 2024-10-04

**Authors:** Larissa L.S. Scholte, Justin Browne, David J. Nolan, Peyton St. John, Katherine Tracy, Rafaela S. Thur, Ghangzhao Li, Susanna L. Lamers, Paige Bracci, Michael S. McGrath, Jeffrey M. Bethony

**Affiliations:** 1 Department of Microbiology, Immunology and Tropical Medicine, The George Washington University, Washington, DC 20052, USA; 2 Bioinfoexperts, LLC, Thibodaux, LA 70301, USA; 3 Department of Epidemiology and Biostatistics, University of California, San Francisco, San Francisco, CA 94158, USA; 4 Department of Laboratory Medicine, Pathology, and Medicine, University of California, San Francisco, San Francisco, CA 94110, USA

**Keywords:** Cancer, Sequencing, Molecular Biology, Gene Expression

## Abstract

Kaposi sarcoma (KS) punch biopsies present unique challenges for extracting nucleic acids, which can be exacerbated by their long-term stabilization in RNAlater. Here, we present a protocol for simultaneously isolating DNA, RNA, and miRNA from a single KS punch biopsy. We detail the steps for preparing reagents and supplies, disrupting KS tissue using manual and mechanical methods, isolating DNA and total RNA, evaluating nucleic acid quality, and storing nucleic acids long-term.

## Highlights

• Instructions for tissue disruption of challenging/recalcitrant samples
• Protocol for the simultaneous isolation of DNA and total RNA
• Optimized steps for achieving higher and more consistent nucleic acid yields
• Quality control strategy for assessing DNA/RNA concentration and integrity

## Abstract (editor highlights)

Publisher’s note: Undertaking any experimental protocol requires adherence to local institutional guidelines for laboratory safety and ethics.

## Graphical abstract

**Figure:**
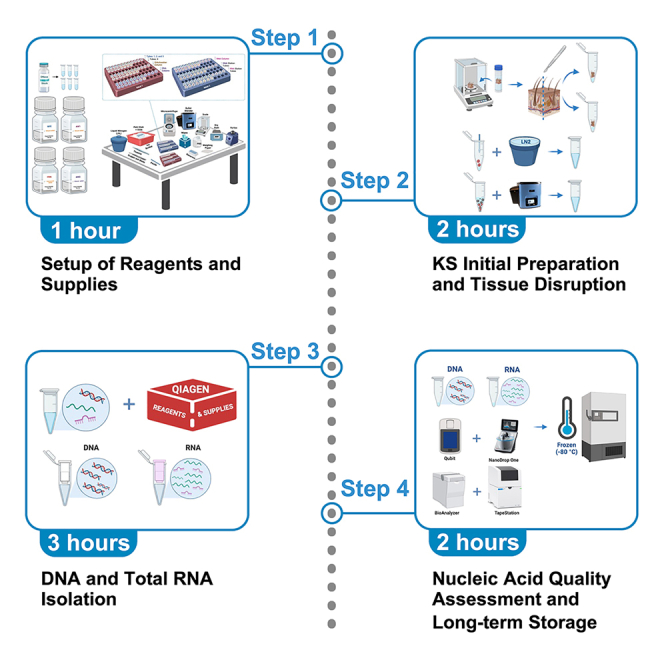

## Before you begin

This protocol describes the simultaneous isolation of DNA, RNA, and miRNA from Kaposi Sarcoma biopsies stored in RNAlater in the AIDS and Cancer Specimen Resource (ACSR).^1^ These samples were collected from patients enrolled in the Antiretrovirals for Kaposi Sarcoma (ARKS) study.^2^

Kaposi Sarcoma (KS) is the most common cancer in people with Human Immunodeficiency Viruses (PWH). KS results in multifocal angioproliferative tumors, which typically manifest as skin lesions, although mucosal and visceral lesions may also occur.^3^^,^^4^ The underlying cause of HIV-associated KS is infection with human herpesvirus 8 (HHV-8), an oncogenic double-stranded DNA virus known as the Kaposi Sarcoma-related herpesvirus (KSHV).^5^

Biopsies taken from KS lesions can be challenging for multiple genomic and transcriptomic analyses due to difficulties in tissue homogenization and nucleic acid yields. The tumor’s enhanced vascularity combined with the extravasation of erythrocytes and hemosiderin deposits, result in the characteristic dark and hemorrhagic nature of KS tumors.^6^ Additionally, the increased presence of basement membrane components contributes to the recalcitrant nature of KS biopsies. This challenge becomes more pronounced when KS samples have been stabilized in high salt buffers like RNAlater and stored for long periods.

Herein, we present an optimized protocol for KS biopsies, which may also be applied to other difficult-to-lyse tissues to maximize nucleic acid yields and potential downstream applications. The protocol is derived from the comparison of two commercially available kits - NucleoSpin TriPrep and AllPrep DNA/RNA/miRNA Universal - which allow for the simultaneous extraction of DNA and RNA from the same biological sample. Before conducting tests on human KS samples, the protocol was first tested and validated using mouse tissue.

The NucleoSpin TriPrep kit, manufactured by Macherey-Nagel, is a convenient one-column preparation method that allows for simultaneous high-quality DNA/RNA isolation. It is a faster protocol than the AllPrep DNA/RNA/miRNA by QIAGEN and allows for similar amounts of starting material. However, once RNA and DNA are bound to the same silica membrane, DNA binding capacity strongly depends on the amount of RNA and the small RNA fraction is lost. Furthermore, DNA elution is performed under selective conditions to avoid RNA elution. Depending on the elution buffer temperature, RNA may be partially eluted during DNA recovery. Finally, NucleoSpin columns were easily obstructed, requiring additional centrifugation steps and ultimately delivering poor, inconsistent nucleic acid yields. On the other hand, the AllPrep DNA/RNA/miRNA Universal kit, is designed to simultaneously purify nucleic acids from difficult-to-lyse tissues using a two-column strategy. This kit requires a longer processing time but provides higher and more consistent yields while also recovering the miRNA fraction. This kit tends to provide reduced 260/230 ratios, but the QIAGEN Research & Development team showed that contaminations with guanidine thiocyanate of up to 100 mM in RNA samples do not compromise the reliability of amplifications in downstream applications.^7^ This finding was confirmed during the protocol optimization steps. Thus, the presented protocol results from the optimization of the disruption steps in combination with a modified version of the AllPrep DNA/RNA/miRNA Universal kit protocol using an alternative lysis buffer (i.e., Buffer RLT by QIAGEN) and additional washing steps to improve purity ratios. This optimized strategy maximizes the number of studies that can be performed on rare, fresh, or archival biopsies.

**Figure 1:**
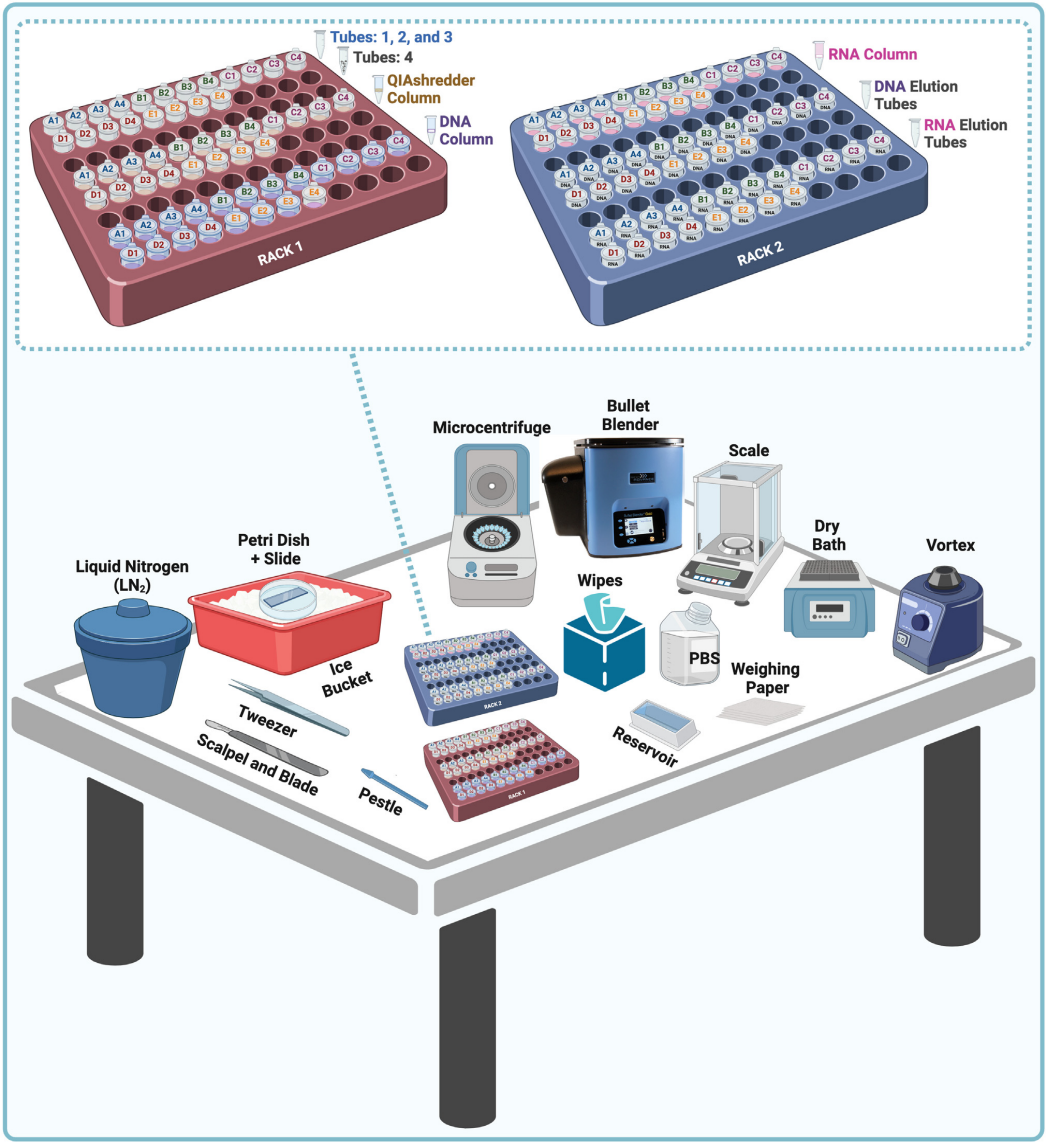
Overview of the bench setup for sample processing, including equipment utilized during nucleic acid isolation

### Preparation of DNase stock I

**Timing: 10 min**

1. Using an RNase-free needle and syringe, inject 550 μL of RNase-free water into the lyophilized DNase I vial. Do not open the vial containing the DNase Stock I. It may cause loss of lyophilized DNase I.
2. Gently invert the vial to mix the water and DNase I. Do not vortex the vial. DNase I is sensitive to physical denaturation. If insoluble material is observed, it will not affect the DNase I performance.
3. Prepare single use aliquots in labeled vials and store them at −30°C to −15°C for up to 9 months.

***Note:*** Do not refreeze DNase I aliquots. Thawed aliquots can be stored at 2°C–8°C for up to 6 weeks.

### Preparing buffer FRN

**Timing: 5 min**

4. Add 42 mL of molecular biology grade isopropanol to the 14 mL Buffer FRN concentrate container. In case a precipitate is observed, dissolve it by warming the buffer at 37°C with gentle agitation for 5 min. Add the isopropanol after equilibration to ambient temperature (18°C–25°C).
5. On the FRN bottle label, record the date, check the box to indicate isopropanol addition, and store the container at ambient temperature (18°C–25°C).

### Preparation of buffer RPE

**Timing: 5 min**

6. Add 44 mL of molecular biology grade ethanol (96%–100%) to the 11 mL Buffer RPE concentrate container.
7. On the RPE bottle label, record the date, check the box to indicate ethanol has been added, and store the container at room temperature (18°C–25°C).

### Preparation of buffer AW1

**Timing: 5 min**

8. Add 25 mL of molecular biology grade ethanol (96%–100%) to the 19 mL Buffer AW1 concentrate container.
9. On the AW1 bottle label, note the date, mark the checkbox to confirm the addition of ethanol, and store the container at ambient temperature (18°C–25°C).

### Preparation of buffer AW2

**Timing: 5 min**

10. Add 30 mL of molecular biology grade ethanol (96%–100%) to the bottle containing 13 mL Buffer AW2 concentrate container.
11. On the AW2 bottle label, write the date, check the box to indicate ethanol has been added, and store the container at room temperature (18°C–25°C).

### Preparation of buffer RLT (lysis buffer)

**Timing: 10 min**

12. Label a 15 mL conical tube as “RLT Buffer + ß-ME” and calculate the total volume of lysis buffer that will be required according to the number of samples being processed: 600 μL of Buffer RLT is required per vial. The example below is for 20 preparations, considering that: i) a maximum of five samples is recommended to be processed on the same day; ii) each sample will be processed in a total of four vials, and; iii) two extra volumes are added to account for buffer used during manual disruption and volume lost due to pipetting error. If a different number of samples will be processed, adjust accordingly.

Example:[5samples×4(multiplehomogenizationrounds)]+2(extravol.)×600μL=13,200μL

**CRITICAL:** This protocol utilizes “*Buffer RLT*” instead of “*Buffer RLT Plus*” (usually recommended for hard-to-lyse samples). Tests performed during the optimization steps showed that Buffer RLT Plus did not perform well on KS skin biopsies stored for decades in RNAlater.

13. Add 13,200 μL of Buffer RLT to a 15 mL conical Tube. In case a precipitate is observed in the RLT, dissolve it by warming at 37°C with gentle agitation for 5 min and add ß-ME after equilibration to ambient temperature (18°C–25°C).
14. Calculate the total of ß-ME that will be required according to the number of samples being processed: 6 μL of ß-ME is required per vial.
15. Add 132 μL of ß-ME to the same conical tube, homogenize well using a 15 mL serological pipette, and store the container at ambient temperature (18°C–25°C) for up to a month. Although the RLT Buffer + ß-ME solution is stable for up to a month at ambient temperature, if possible, prepare a fresh solution prior to each processing session.

**CRITICAL:** ß-ME is a toxic reducing agent that will denature RNases by changing protein conformation and result in enzymatic inactivity. Always use a chemical fume hood and required personal protective equipment (PPE) when manipulating the ß-ME stock bottle.

### Setup of supplies

**Timing: 30 min for a total of five samples**

16. Clean the bench and equipment with ethanol 70% and RNase AWAY.
17. Use two clean 96-well microtube racks to prepare vials for DNA and RNA processing.
18. Label two sets of four 1.5 mL safe-lock microcentrifuge tubes per sample, such as: A1, A2, A3, A4; B1, B2, B3, B4; C1, C2, C3, C4; D1, D2, D3, D4, and; E1, E2, E3, E4.

***Note:*** The first set will be utilized for tissue disruption and the second set for nucleic acid elution.

**CRITICAL:** Ideally, sample processing should be performed by two operators. A single operator should not process more than five samples at a time. Total processing time can take up to eight hours with short breaks.

19. To the fourth safe-lock microcentrifuge tube for each sample (e.g., A4, B4, C4, D4, and E4), add:
  a. One scoop (∼100 μL) of 0.9–2 mm RNase-free SST beads, and;
  b. One scoop (∼100 μL) of 3.2 mm RNase-free SST beads.

***Alternatives:*** In case a scoop is not available, weigh the beads and add a total of 550 mg of 0.9–2 mm and 550 mg of 3.2 mm (or five units) of RNase-free SST beads to the fourth vial of each sample.

**CRITICAL:** Stainless steel beads have higher density and are required due to the recalcitrant nature of KS samples. The volume of beads may be equal or greater than 50% of the lysis buffer volume. Using less beads may lead to poor tissue disruption.

20. Label one set of four 1.5 mL QIAshredder spin columns in 2 mL collection tubes per sample using the same sample code described in step 18. To avoid mixing samples, label both the column lid and the collection tube.

***Note:*** The QIAshredder column will be utilized to homogenize the sample, by reducing viscosity and removing tissue fragments that may obstruct the DNA and RNeasy columns.

21. Label one set of four 1.5 mL DNA Mini Spin columns in 2 mL collection tubes per sample (purple ring) using the same sample code described in step 18. To avoid mixing samples, label both the column lid and the collection tube.
22. Label one set of four 1.5 mL RNeasy Mini Spin columns in 2 mL collection tubes per sample (pink column) using the same sample code described in step 18. To avoid mixing samples, label both the column lid and the collection tube.
23. For each sample, label a sterile petri dish, a sterile microscope slide, and a sterile reservoir.

**Pause point:** To reduce the processing total time, the setup of supplies (steps 16–23) may be performed up to 16 hours prior to sample processing (e.g., in the evening of the day prior) if the columns are maintained in a temperature-controlled environment (18°C–25°C). Alternatively, it may also be performed right before sample processing.

24. Prepare the processing bench with necessary supplies: high precision sterile scalpel blades, sterile scalpels, sterile forceps, pestles, weighing paper, laboratory wipes, sterile reservoirs, cold PBS, a filled insulated ice container, and an insulated Dewar filled with at least one liter of LN_2_. Figure 1 shows an overview of the bench setup for sample processing.

***Note:*** The ice container needs to be large enough to fit a disposable sterile petri dish with additional room for up to twenty 1.5 mL safe-lock microcentrifuge tubes.

**CRITICAL:** Liquid nitrogen (LN_2_) is a cryogenic fluid that can cause severe bodily harm such as eye damage, frostbite injuries, and burns, which may occur within seconds of exposure. Never handle LN_2_ without following safety precautions and wearing required personal protective equipment (PPE: closed-toed shoes, long pants, cryoprotecting gloves, face shield, and lab coat). Do not use or store LN_2_ in rooms without ventilation since it may cause an oxygen-deficient atmosphere.

### Institutional permissions

Biospecimens from the ACSR were collected after obtaining institutional review board (IRB) approval for the ARKS source clinical protocol and informed consent from enrolled patients. ACSR samples are provided as a service to the research community and shall be utilized for research purposes only.

## Key resources table

**Table:** 

REAGENT or RESOURCE	SOURCE	IDENTIFIER
**Chemicals, peptides, and recombinant proteins**		

1× PBS (sterile), Ca^2+^Mg^2+^ free	Gibco	Cat.# 10010-023 or equivalent
1.5 mL Safe-Lock microcentrifuge tubes	Eppendorf	Cat.# 0030120094
AllPrep DNA/RNA/miRNA Kit	Qiagen	Cat# 80224
β-Mercaptoethanol (β-ME, 14.3 M)	EMD Millipore	Cat# 444203 or equivalent
Buffer AW2 (concentrate)^a^	QIAGEN	Cat# 19072
Buffer RLT^b^	QIAGEN	Cat# 79216
Buffer RPE (concentrate)^c^	QIAGEN	Cat# 1018013
Ethanol (96%–100%)	Fisher	Cat# BP2818-500 or equivalent
Isopropanol (99.9%)	Thermo Scientific	Cat# T03618-1000 or equivalent
Microcide SQ	Hamilton Company	Cat# 3995-01 or equivalent

**Other**		

Forceps (disposable)	Burkle	Cat# 5378-1040 or equivalent
Microscope slides	Fisher	Cat# 12-550-A3 or equivalent
Needle	BD	Cat# BD305127 or equivalent
Pestle	Fisher	Cat# 12-141-364 or equivalent
QIAshredder	QIAGEN	Cat# 79656
Stainless steel beads 3.2 mm	Next Advance	Cat# SSB32-RNA or equivalent
Stainless steel beads 0.9–2.0 mm	Next Advance	Cat# SSB14B-RNA or equivalent
Sterile, RNase-free pipet tips 10 μL	Neptune	Cat# BT10 or equivalent
Sterile, RNase-free pipet tips 200 μL	Neptune	Cat# BT200 or equivalent
Sterile, RNase-free pipet tips 1,000 μL	Neptune	Cat# BT1000 or equivalent
Surgical blades	Integra	Cat# 4–110 or equivalent
Syringe	BD	Cat# BD-309628 or equivalent
Weighing paper	Fisher	Cat# 09-898-12A or equivalent
2100 BioAnalyzer	Agilent	Cat# Version 2100 or equivalent
Bench mini microcentrifuge	Corning	Cat# 6770 or equivalent
Bullet blender	Next Advance	Bullet Blender Gold or equivalent
Dry bath or heating block	Fisher	Cat# S29028 or equivalent
Microcentrifuge	Eppendorf	Cat# 5424 or equivalent
Qubit	Thermo Fisher Scientific	Cat# Version 4 or equivalent
TapeStation	Agilent	Cat# Version 4150 or equivalent
Vortex	OHAUS	Cat# 30392115 or equivalent

a An extra bottle of Buffer AW2 is required. The protocol includes an extra washing step.

b Utilized to replace the Buffer RLT Plus that comes with the AllPrep DNA/RNA/miRNA kit.

c An extra bottle of Buffer RPE is required. The protocol includes an extra washing step.

## Materials and equipment

**Table:** DNase Stock I Solution^a^

Reagent	Final concentration	Amount
Lyophilized DNase Stock I	2.7 Kunitz units	1500 Kunitz units
RNase-free water	N/A	550 μL
Total	N/A	550 μL

^a^Stored at −30 to −15°C for up to 9 months.

**Table:** Buffer FRN^a^

Reagent	Final concentration	Amount
Buffer FRN (concentrate)^b^	25% (v/v)	14 mL
Isopropanol	75% (v/v)	42 mL
Total	N/A	56 mL

^a^Stored at 18°C–25°C for up to 9 months after reagent delivery.

^b^QIAGEN Proprietary Information.

**Table:** Buffer RPE^a^

Reagent	Final concentration	Amount
Buffer RPE (concentrate)^b^	20% (v/v)	11 mL
Ethanol	80% (v/v)	44 mL
Total	N/A	55 mL

^a^Stored at 18°C–25°C for up to 9 months after reagent delivery.

^b^QIAGEN Proprietary Information.

**Table:** Buffer AW1^a^

Reagent	Final concentration	Amount
Buffer AW1 (concentrate)^b^	43% (v/v)	19 mL
Ethanol	57% (v/v)	25 mL
Total	N/A	44 mL

^a^Stored at 18°C–25°C for up to 9 months after reagent delivery.

^b^QIAGEN Proprietary Information.

**Table:** Buffer AW2^a^

Reagent	Final concentration	Amount
Buffer AW2 (concentrate)^b^	30% (v/v)	13 mL
Ethanol	70% (v/v)	30 mL
Total	N/A	43 mL

^a^Stored at 18°C–25°C for up to 9 months after reagent delivery.

^b^QIAGEN Proprietary Information.

**Table:** Buffer RLT (Lysis Buffer)

Reagent	Final concentration	Amount
**Buffer RLT^b^**	99% (v/v)	13,200 μL
**ß-Mercaptoethanol (ß-ME)**	1% (v/v)	132 μL
Total	N/A	13,332 μL

^a^Stored at 18°C–25°C for up to a month.

^b^QIAGEN Proprietary Information.

## Step-by-step method details

**Figure 2:**
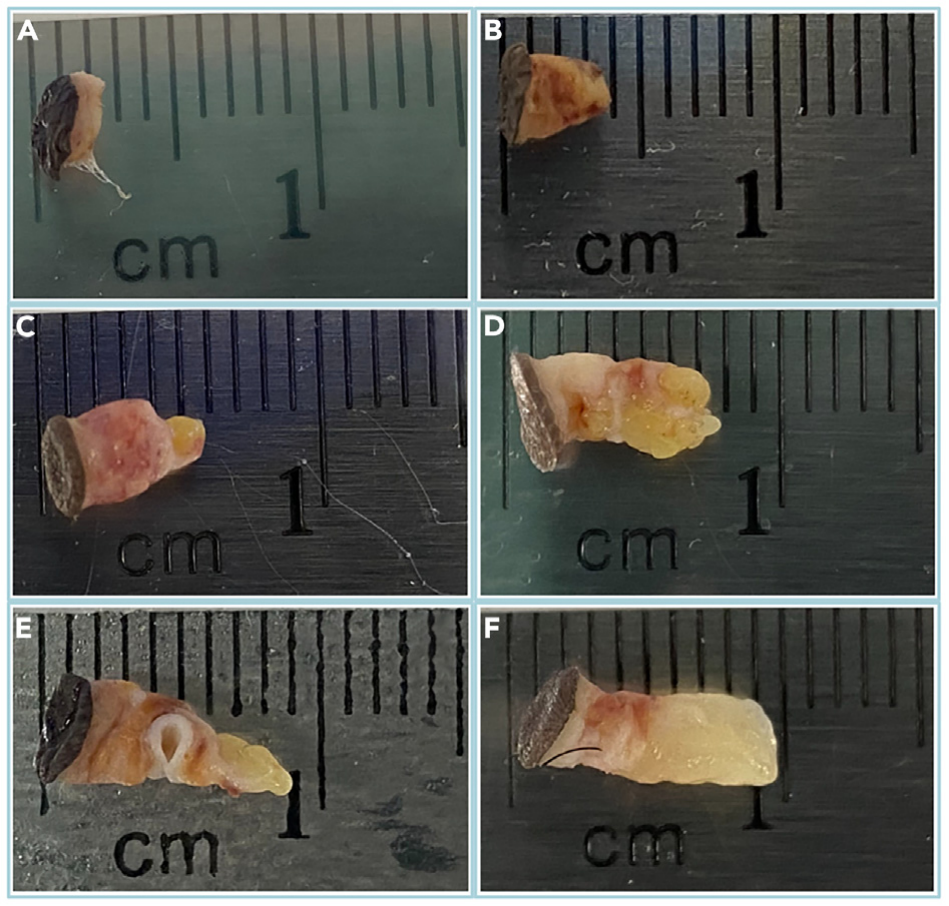
Representative ARKS samples exhibiting the distinct biopsy sizes and tissue composition (A) shows the smallest KS biopsy processed (5.7 mg), (B–E) illustrate the biospecimen diversity in terms of dimensions and anatomical composition, and (F) shows the largest KS biopsy processed (56.2 mg).

A total of 136 histologically confirmed KS biopsies were collected from Kaposi Sarcoma lesions using a 3 mm cylindrical single-use punch from May 2007 to February 2011 (Figure 2). All biopsies were stabilized in RNAlater overnight at +2°C–8°C prior to freezing at −80°C for long-term storage.

### KS sample initial preparation

**Timing: 30 min**

**Figure 3:**
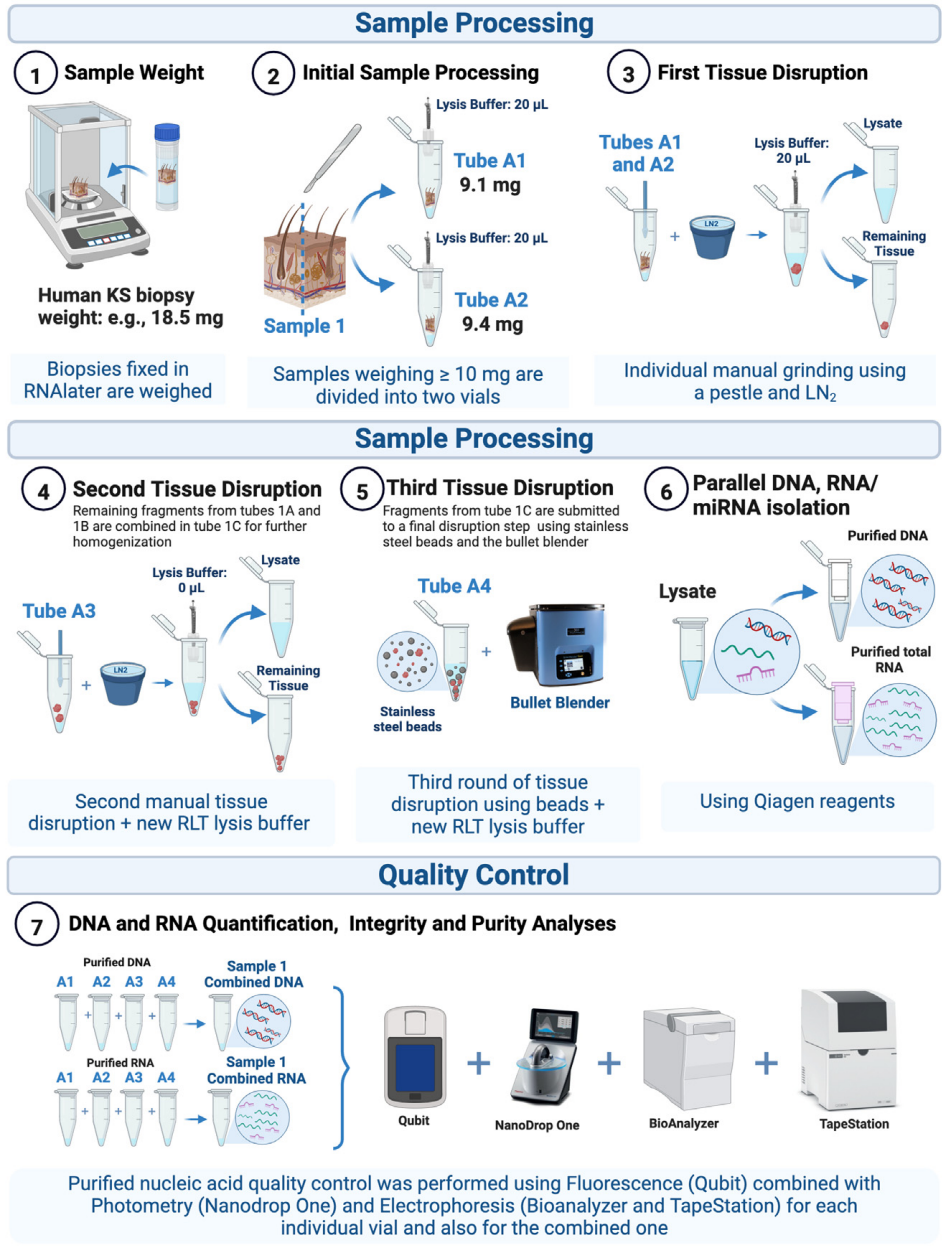
Workflow summarizing the tissue disruption, DNA and total RNA isolation, and quality assessment steps

The following steps provide instructions on initial biopsy handling in preparation for subsequent tissue disruption and processing for nucleic acid isolation. Figure 3 summarizes the complete workflow, from KS initial preparation until DNA/RNA quality assessment.

1. Add 20 μL of RLT Buffer + ß-ME to the first three 1.5 mL safe-lock microcentrifuge tubes for each sample (e.g., A1, A2, and A3).

***Note:*** It will facilitate manual disruption.

2. Add 400 μL of RLT Buffer + ß-ME to the fourth 1.5 mL safe-lock microcentrifuge tube for each sample (the tube containing SST beads – e.g., A4).
3. Thaw the stabilized KS biopsies on ice.

**CRITICAL:** Thawed samples must be immediately processed or kept at 2°C–8°C.

4. Using sterile forceps, carefully remove the KS biopsy from the vial and quickly wash it in a sterile disposable reservoir containing cold sterile PBS to remove excessive salt from the RNAlater solution.
5. Quickly and gently dry the tissue using a lab wipe and transfer it to a tared analytical scale on top of a clean sheet of weighing paper.
6. After recording the tissue weight, transfer biopsies weighing >10 mg to a clean microscope slide on a sterile petri dish placed on ice.
  a. Cut it into two (approximately equal) portions using a sterile blade.
  b. Transfer portion one to the first vial containing 20 μL of RLT Buffer + ß-ME (e.g., A1).
  c. Transfer portion two to the second vial also containing 20 μL of RLT Buffer + ß-ME (e.g., A2).

***Alternatives:*** Tissue samples weighing ≤ 10 mg should be transferred directly to the first safe-lock vial containing 20 μL of RLT Buffer + ß-ME (e.g., A1). In this case, the second vial can be discarded (e.g., A2).

7. Place the vials (e.g., A1 and A2) on ice until tissue disruption is initiated.
8. Replace all disposable items and repeat steps 4 through 7 for each additional sample (e.g., B, C, D, and E). Use new forceps, lab wipes, weighing paper sheets, blades, petri dishes, microscope slides, and reservoirs filled with cold PBS for each sample being processed.

**CRITICAL:** If not using disposable items, make sure that sets of appropriately cleaned sterile supplies are available for each sample.

### Sample disruption

**Timing: 1 h and 30 min**

Efficient disruption and cell lysis of the starting material are well-known requirements for successful simultaneous DNA and RNA isolation with high quality nucleic acid yields. Long and thin shaped tissue samples tend to homogenize more efficiently than cubic or round shaped samples. To achieve thorough homogenization and avoid unwanted changes in the gene expression profile, always refreeze thawing tissue in LN_2_ during the disruption process, while keeping thawed samples on ice.

9. Use a new disposable pestle and LN_2_ to manually grind the tissue placed in the first vial of each sample for about 5–10 min (e.g., A1, B1, C1, D1, E1).

**CRITICAL:** Be careful not to apply excessive pressure to the tissue if it is completely frozen, as it might be forced out of the tube, risking sample loss.

***Alternatives:*** P1000 tips may also be utilized for tissue disruption. In case the tip is obstructed with tissue or dented, use a sterile needle to unobstruct or straighten it.

10. Once tissue homogenization is completed, add 600 μL of RLT Buffer + ß-ME into the vial. As the buffer is added, rinse the pestle or pipette tip used for tissue disruption.
11. Close the safe-lock vial and quickly vortex the tube at maximum speed.
12. Quick-spin the tube at 20,000g for 1 min, collect the lysis buffer avoiding the tissue fragments and transfer it to the corresponding QIAshedder column.
13. Quickly freeze the remaining tissue fragments and transfer them into the third tube (e.g., A3) using a new P1000 tip. Keep the tube on ice.
14. For the second vial of each sample (e.g., A2), repeat steps 9 through 13.

***Note:*** Once this step is completed, tissue fragments from the first and second tubes (e.g., A1 and A2) will have been combined into the third tube (e.g., A3).

15. Repeat steps 9 through 12 for the third vial of each sample (e.g., A3).
16. Quickly freeze the remaining tissue fragments from the third tube (e.g., A3) and transfer them into the fourth tube (e.g., A4) using a new P1000 tip.

***Note:*** Tube 4 should already contain the SST beads and 400 μL of RLT Buffer + ß-ME.

17. Submit the fourth tube (e.g., A4) to mechanical disruption in the Bullet Blender at speed 6 for 2 min.

**CRITICAL:** Make sure to use safe-lock vials for mechanical homogenization. Using different tubes may result in sample loss due to opening or breaking during agitation.

***Alternatives:*** If using alternative bullet blenders or bead beaters, the ideal speed and disruption duration may need to be optimized to avoid excessive DNA shearing (if the length of the purified DNA is shorter than 15 kb–30 kb). It is recommended to start at 50% maximum speed for 2 min, and increase the speed gradually as needed.

18. Once tissue homogenization is completed, centrifuge the tube at 20,000 × *g* for 1 min, collect the lysis buffer while avoiding tissue fragments and transfer it to the corresponding labeled QIAshedder column.
19. Rinse the beads with an additional 200 μL of RLT Buffer + ß-ME and transfer the lysis buffer to the same corresponding QIAshedder column. If needed, re-spin the tube at 20,000 × *g* for 30 s to 1 min and collect the remaining RLT Buffer + ß-ME.

***Note:*** In total, about 600 μL of lysis buffer is expected to be recovered for the fourth tube (e.g., A4). If a volume lower than that is obtained, re-spin the tube and use a long 200 μL pipette tip to improve recovery.

### DNA isolation

**Timing: 1 h**

This section describes the necessary steps to ensure appropriate DNA recovery from the KS lysate. DNA isolation steps should be performed at ambient temperature (18°C–25°C) and the operators should complete the processing steps as quickly as possible.

20. Centrifuge the collection tubes containing the QIAshredder columns at 20,000 × *g* for 2 min.

***Note:*** The lysate is homogenized as it passes through the biopolymer-shredding column by removing insoluble material and reducing lysate viscosity.

21. Remove the QIAShredder column from the collection tube and transfer the homogenized lysate to the corresponding DNA Mini Spin Column placed in a 2 mL collection tube.

**CRITICAL:** Be careful not to disturb the pellet that may be present in the QIAShredder collection tubes. This pellet contains stainless steel microparticles resulting from the collision between beads during sample mechanical disruption. If transferred to the DNA Mini Spin Column, the silica membrane may become obstructed, reducing its performance.

22. Close the DNA Mini Spin Column and centrifuge for 30 s at 20,000 × *g.*
23. Transfer the DNA Mini spin columns containing bound DNA to a new labeled 2 mL collection tube.
  a. Do not discard the collection tubes containing the flow-through.
  b. Use the lids provided in the QIAShredder column kit to close them. The flow-through from this step will be utilized for RNA purification in step 40.

***Note:*** Make sure the collection tube containing the flow-through is labeled before you transfer the DNA column into a new labeled 2 mL microcentrifuge tubes.

24. If a single operator is processing the samples, store the DNA Mini Spin Column at ambient temperature (18°C–25°C), under refrigeration (2°C–8°C), or on wet ice for later DNA purification steps.

**CRITICAL:** Do not freeze the columns. Proceed with DNA isolation as soon as RNA extraction is completed.

***Alternatives:*** If two operators are processing the samples, one must proceed with DNA isolation (step 25) whilst the other conducts the RNA extraction (step 40).

25. Add 350 μL of Buffer AW1 to the AllPrep DNA Mini spin column from step 24.
  a. Close the lid and centrifuge for 30 s at 20,000 × g to wash the column membrane.
  b. Discard the flow-through, dry the collection tube rim using a clean lab wipe, and reuse the collection tube in step 27.
26. Label a 2 mL tube as “Proteinase K Incubation Mix”.
  a. Calculate the total volume required according to the number of samples being processed: 20 μL of Proteinase K + 60 μL of Buffer AW1 per vial.
  b. Add two extra volumes to account for buffer lost due to pipetting error.
  c. Mix well by gently pipetting.

***Note:*** Example: if processing 20 vials resulting from five initial samples, prepare the incubation mix for a total of 22 samples

(22×20μLofProteinaseK)+(22×60μLofBufferAW1)=1,760μL

27. Add 80 μL of the “Proteinase K Mix” to the center of the column membrane and keep the columns on the benchtop at ambient temperature (18°C–25°C) for 5 min.
28. During the 5 min break, label two 1.5 mL tubes as “Buffer EB”.
29. Calculate the total volume needed for DNA elution based on the number of samples: 105 μL per vial.
  a. Include two extra volumes to account for loss due to pipetting error.
  b. Split the total volume into the two labeled 1.5 mL vials.

***Note:*** Example: if processing 20 vials resulting from five initial samples, aliquot Buffer EB for a total of 22 samples,

22×105μLofBufferEB=2,310μL

30. Place the vials containing Buffer EB in a dry bath at 70°C. Warm Buffer EB will be required for elution (steps 35 and 37).

***Note:*** Heating the Buffer EB may improve the total DNA yield as it facilitates the release of column membrane bound DNA.

31. To wash the column, add 350 μL of Buffer AW1 to the AllPrep DNA Mini spin column.
  a. Close the lid and centrifuge for 30 s at 20,000 × g.
  b. Discard the flow-through.
  c. Dry the column rim using a clean lab wipe, and reuse the collection tube in step 32.
32. Add 500 μL of Buffer AW2 to the AllPrep DNA Mini spin column.
  a. Close the lid and centrifuge for 30 s at 20,000 × g.
  b. Discard the flow-through.
  c. Dry the column rim using a clean lab wipe, and reuse the collection tube in step 32.
33. Repeat step 32 to further reduce the salt concentration, improve elution, and obtain higher eluate purity.
34. Centrifuge the columns once again for 1 min at 20,000 × g to further dry the spin column membrane.

***Note:*** This reduces the chances of residual ethanol being carried over during elution which will potentially impact downstream applications.

35. Carefully transfer the AllPrep DNA Mini spin column to a new labeled 1.5 mL safe-lock microcentrifuge tube.
  a. Add 55 μL of Buffer EB pre-heated at 70°C to the center of the spin column membrane.
  b. Close the column lid and incubate at room temperature for 5–10 min.

***Note:*** A total of 55 μL is utilized for elution instead of 50, accounting for the approximate 5 μL usually absorbed by the spin column membrane.

36. Centrifuge for 1 min at 20,000 × g to elute the DNA.

***Note:*** At this step centrifugation is performed with the column lid closed, but with the collection tube lid open. Make sure to properly clean equipment with ethanol 70% prior to use.

***Note:*** The AllPrep DNA/RNA/miRNA kit provides regular 1.5 mL microcentrifuge tubes for elution. However, it is advisable to use the safe-lock tubes since they are less prone to breaking during centrifugation with the lid open.

37. Add additional 50 μL of Buffer EB pre-heated at 70°C to the center of the spin column membrane to further elute DNA using the same collection tube.

***Note:*** The final eluate volume should be approximately 100 μL per vial.

38. Eluates from the same sample (e.g., A1, A2, A3, and A4) can be combined immediately for quality assessment or quality control can be performed for each vial independently.
39. For long-term storage, freeze at −80°C.

### TOTAL RNA isolation

**Timing: 1 h and 30 min**

This section details the steps for isolating total RNA. Sample refrigeration is not required. All steps should be performed at room temperature (18°C–25°C) and the technicians should work as quickly as possible, avoiding pauses longer than those defined in the protocol. To avoid compromising the RNA integrity due to the omnipresence of RNases, work in a RNase-free environment: follow good practices for aseptic technique; change gloves whenever touching surfaces and equipment that were not cleaned and decontaminated with RNase AWAY; keep tubes closed whenever possible, and keep purified RNA on ice until long-term storage at −80°C.

40. Add 80 μL of Proteinase K to the collection tube containing the flow-through from step 23. Mix well by pipetting.
41. To the same tube, add 350 μL of 96%–100% of molecular biology grade ethanol, mix well by pipetting, and incubate for 10 min at room temperature.
42. During the 10 min break, label a 1.5 mL tube as “RNase-free H_2_O” and calculate the total volume required for RNA elution according to the number of samples being processed: 55 μL per vial. Add two extra volumes to account for loss due to pipetting error.

***Note:*** Example: if processing 20 vials resulting from five initial samples, aliquot RNase-free H_2_0 for a total of 22 samples,

$$22\times 55\;\mu L\;\text{of}\;\text{RNase}-\text{free}\;H_{2}O=1,210\;\mu L$$

43. Place the vial containing RNase-free H_2_0 in a dry bath at 70°C. Warm RNase-free H_2_0 will be required for RNA elution (step 58).

***Note:*** Heating the RNase-free H_2_0 may improve the RNA total yield as it facilitates the release of column membrane bound RNA.

44. Add 750 μL of 96%–100% of molecular biology grade ethanol to the collection tube and mix well by pipetting.
45. Transfer 600 μL of the sample to the corresponding RNeasy Mini spin column placed in a labeled 2 mL collection tube.
  a. Carefully close the column lid and centrifuge for 30 s at 20,000 × g.
  b. Discard the flow-through, dry the column rim using a clean lab wipe, and reuse the collection tube in step 46.

***Note:*** Proceed with the transfer to the column even if visible precipitates may have formed.

46. Repeat step 45 until the entire sample has passed though the RNeasy Mini spin column. Mix well by pipetting before each transfer. A total of three passes are required to pass the whole sample volume through the column.
47. Add 500 μL of Buffer RPE to the RNeasy Mini spin column to reduce salt concentration and provide ideal conditions for the DNase I activity.
  a. Carefully close the column lid and centrifuge for 30 s at 20,000 × g.
  b. Discard the flow-through.
  c. Dry the column rim using a clean lab wipe, and reuse the collection tube in step 48.
48. Label a 2 mL tube as “DNase I Incubation Mix”.
  a. Calculate the total volume required according to the number of samples being processed: 10 μL of reconstituted DNase I + 70 μL of Buffer RDD per vial.
  b. Add two extra volumes to account for buffer lost due to pipetting error.
  c. Mix well by gently pipetting.

Example: If processing 20 vials resulting from five initial samples, prepare the incubation mix for a total of 22 samples

Example:(22×10μLofDNase)+(22×70μLofBufferRDD)=1,760μL

**CRITICAL:** DNase I is sensitive to physical denaturation. Do not vortex the vial.

49. Add 80 μL of the DNase I incubation mix to the center of the column membrane and keep the columns on the benchtop at ambient temperature (18°C–25°C) for 15 min.
50. Add 500 μL of ready-to-use Buffer FRN to the RNeasy Mini spin column.
  a. Close the lid gently and centrifuge it for 30 s at 20,000 × g.
  b. Save the flow-through for use in step 51.

**CRITICAL:** Buffer FRN has strong binding capacity and will optimize the total RNA recovery by creating ideal conditions to rebind small RNA molecules partly released from the column by Buffer RDD – which is required to provide ideal conditions for DNase I activity.

51. Label a new 2 mL collection tube and transfer the RNeasy Mini spin column to the corresponding new vial.
  a. Apply the flow-through from step 50 to the column.
  b. Close the lid and centrifuge for 30 s 20,000 × g.
  c. Discard the flow-through.
  d. Dry the collection tube rim using a clean lab wipe, and reuse the collection tube in step 52.
52. To wash the column, add 500 μL of Buffer RPE to the RNeasy Mini spin column.
  a. Close the column lid and centrifuge for 30 s at 20,000 × g.
  b. Discard the flow-through.
  c. Dry the collection tube rim using a clean lab wipe, and reuse the collection tube in step 53.
53. Repeat step 52 to further reduce the salt concentration for efficient elution and higher eluate purity. Reuse the collection tube in step 54.
54. Add 500 μL of 96%–100% molecular biology grade ethanol to the RNeasy Mini spin column.
  a. Close the lid gently and centrifuge for 30 s at 20,000 × g to wash the column membrane.
  b. Discard the flow-through.
  c. Dry the collection tube rim using a clean lab wipe, and reuse the collection tube in step 55.
55. Repeat washing step 54 and reuse the collection tube in step 56.
56. Centrifuge the closed RNeasy Mini spin column for 1 min at 20,000 × g to ensure that ethanol is not carried over during RNA elution.

***Note:*** Residual ethanol may impact downstream applications.

57. Carefully remove the RNeasy Mini spin column from the collection tube and place it in a new labeled 1.5 mL safe-lock microcentrifuge tube.
58. Add 55 μL of warm RNase-free water pre-heated at 70°C directly to the center of the spin column membrane.
  a. Close the column lid and incubate at ambient temperature for 5–10 min.

***Note:*** A total of 55 μL is utilized for elution instead of 50, accounting for the approximate 5 μL usually absorbed by the spin column membrane.

59. Centrifuge for 1 min at 20,000 × g to elute the total RNA. At this step centrifugation is performed with the column lid closed, but with the collection tube lid open. Thus, make sure the equipment was properly cleaned with ethanol 70% and RNase AWAY.

***Note:*** The AllPrep DNA/RNA/miRNA kit provides regular 1.5 mL microcentrifuge tubes for elution. However, it is advisable to use the safe-lock tubes since they are less prone to breaking during centrifugation with the lid open.

60. Repeat the elution step using the same eluate and collection tube from step 59.

**CRITICAL:** Carefully manipulate the column and the tube at this step.

***Note:*** The final eluate volume should be approximately 50 μL per vial.

61. Eluates from the same sample (e.g., A1, A2, A3, and A4) may be immediately combined for quality assessment or quality control can be performed for each vial independently.
62. Immediately place the eluted RNA on ice and keep it on ice while aliquoting. For long-term storage, freeze at −80°C.

### Nucleic acid quality assessment

**Timing: 1 h and 30 min**

Upon completion of the nucleic acid isolation processes, both DNA and RNA samples were subjected to quantification and integrity analyses using fluorescence- (Qubit 4), photometry- (Nanodrop), and electrophoresis-based methods (2100 Bioanalyzer Instrument and 4150 TapeStation System) following the manufacturer’s guidelines. These assessments provided comprehensive data on the quality and condition of the extracted DNA and total RNA. Besides, Droplet Digital PCR (ddPCR) was utilized to confirm the nucleic acid suitability for downstream analyses.

## Expected outcomes

**Figure 4:**
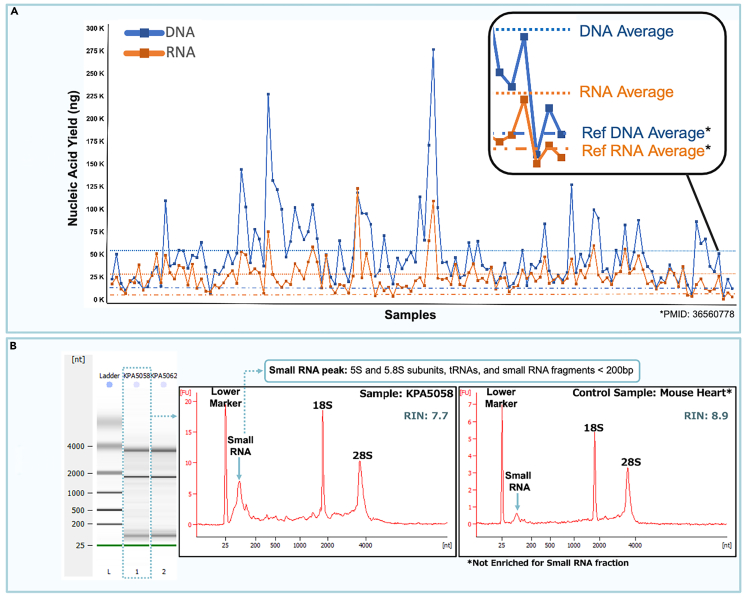
Nucleic acid yield average for 137 processed KS biopsies and RNA electrophoresis data showing miRNA enrichment (A) Obtained total DNA and RNA. (B) RNA Bioanalyzer digital gel image and electropherograms showing the enriched 5S peak (small RNA) resulting from a KS total RNA isolation versus a mouse control containing mRNA only.

Using the protocol described herein, our team processed 137 Kaposi Sarcoma biopsies, weighing from 5.7 to 56.2 mg (average: 26.7 mg). The average DNA yield was 51,260 ng, with DNA integrity numbers (DIN) averaging 6.03 (Figure 4). Meanwhile, the average total RNA yield was 25,714 ng, with RNA integrity numbers (RIN) averaging 8.4. Although the obtained average DIN is lower than the RIN, this result was expected. DNA, being a double-stranded molecule, is more stable than RNA but is more prone to fragmentation during mechanical disruption and silica membrane extraction due to its longer length. Therefore, a DIN of ∼6.0 should be considered a sufficient integrity number in this context. The isolated DNA can be successfully applied to a variety of contemporary downstream assays. Furthermore, the obtained average RIN was unexpectedly high (8.4), considering that the miRNA fraction was also maintained, which impacts the RIN value. Comparing the nucleic yields obtained here with those from Nolan and collaborators (2022),^8^ a greater than 400% increase in DNA yield and an over 800% increase in RNA yield were observed.

Furthermore, the average DNA 260/280 and 260/230 purity ratios obtained using spectrophotometry were 1.85 and 1.60, respectively, while the RNA 260/280 and 260/230 purity ratios were 1.95 and 1.34. Absorbance at these wavelengths is utilized to assess the nucleic acid purity. The 260/280 ratio is related to protein contamination, whereas the 260/230 ratio may indicate the presence of other compounds, including salts. Although optimal DNA and RNA ratios were achieved at 260/280, lower readings at 260/230 were likely due to traces of guanidine thiocyanate – a salt which absorbs strongly at 220–230 nm and is present in a few buffers utilized for nucleic acid extraction. Nonetheless, trace amounts should not impact downstream analyses if the contaminant levels in the final reaction are minimal.

**Figure 5:**
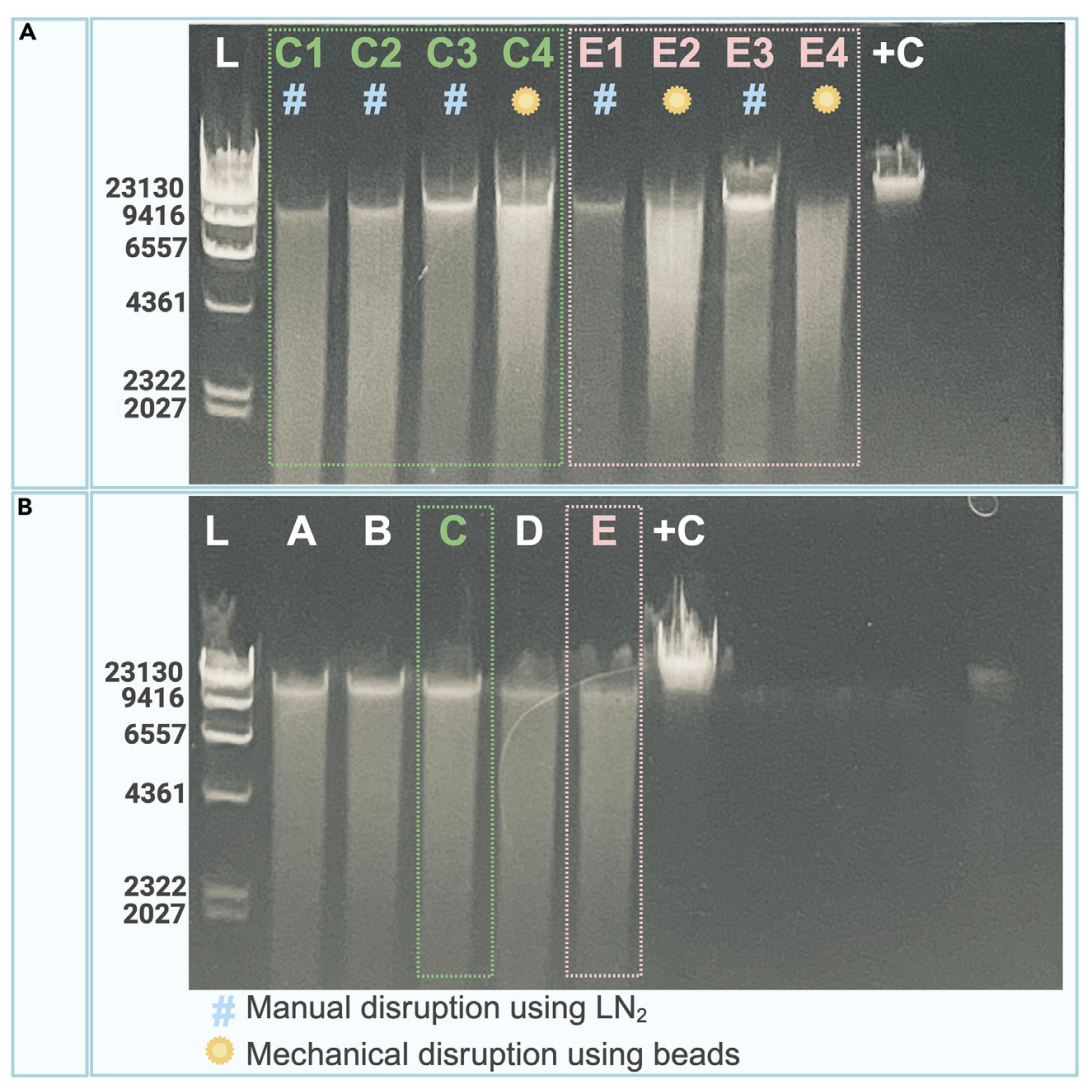
DNA integrity analysis using 0.8% agarose gels (A) Comparison of two Kaposi Sarcoma biopsies (C and E), with similar weight and tissue composition, processed following slightly different disruption processes. Sample C was submitted to three manual disruption steps (C1, C2, and C3) and one mechanical disruption (C4). Sample E was submitted to two manual disruption steps (E1 and E3) and two mechanical disruptions (E2 and E4). The highest molecular weight band corresponds to DNA with higher integrity – fragments of about 10–20 kb – and the smear corresponds to more fragmented DNA – with multiple lengths. (B) Samples submitted mainly to manual disruption show higher integrity (C) than those submitted to mechanical disruption (E).

To achieve the optimal DNA and RNA yields aforementioned, three rounds of tissue disruption were required: two manual and one mechanical (Figure 5). Additional mechanical disruption steps did not improve the total nucleic acid yield and compromised the average integrity.

**Figure 6:**
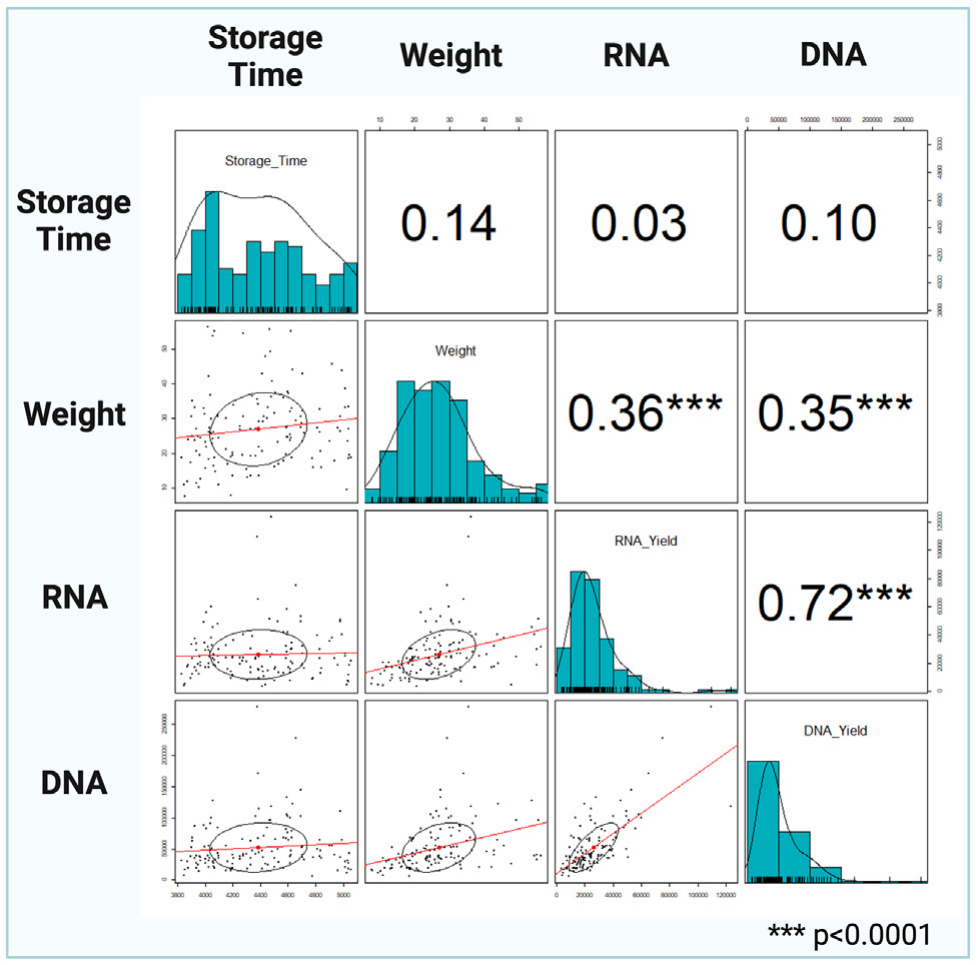
Kaposi Sarcoma biopsy scatter plot matrices comparing four variables: Storage Time, Biopsy Weight, RNA Yield, and DNA Yield Variables are indicated on the diagonal together with histograms showing their distribution. Bivariate scatter plots are presented below the diagonal for variable pairs with red lines fitting linear regression and black lines showing correlation ellipses. The Pearson correlations between the pair of variables are presented above the diagonal with stars (∗∗∗*p* < 0.001) showing the correlation significance.

Findings obtained using the protocol detailed here indicate that nucleic acid yields depend on biopsy size, weight, and tissue composition. Storage time of samples stabilized in RNAlater and maintained at −80°C has no impact on RNA or DNA yield. While weight shows a modest correlation with nucleic acid yields, only DNA and RNA yields are significantly correlated (Figure 6). Large biopsy size and weight may be misleading when considering yield expectations in cases where the majority of the biopsy tissue is composed of fatty tissue, as shown in Figure 2F.

In summary, techniques that enable the simultaneous isolation of DNA, RNA, and miRNA from the same biological sample are particularly valuable when dealing with precious and unique tumor samples stored in biorepositories. Often, having multiple samples from the same patient or a large biopsy allowing for separate DNA and RNA isolations is rare. The protocol developed here not only enhances nucleic acid yields but also enables a simultaneous isolation approach, thereby amplifying the potential for molecular studies that can be performed on a single sample. For instance, KS nucleic acid isolated following this protocol is currently being utilized in KSHV methylation studies to identify subgroups associated with distinct viral loads, lytic viral activity, and patient outcomes. The same samples are pivotal in another study examining biomarkers of progression on antiretroviral therapy, facilitating improved monitoring of individuals at risk for KS progression. Additionally, the Aids and Cancer Specimen Resource (ACSR) retains available DNA and RNA for future research endeavors, opening new frontiers for the development of enhanced diagnostic and monitoring tools, therapeutic treatments, and preventive strategies against Kaposi Sarcoma. The workflow described here extends its utility to other difficult-to-lyse tissues, promising heightened yields and broadening the scope of potential downstream applications.

## Limitations

While the protocol outlined here does not facilitate protein isolation, there is potential for its expansion. The Buffer RLT Plus, originally a component of the AllPrep DNA/RNA/miRNA kit, has been replaced by Buffer RLT. Unlike the former, Buffer RLT lacks detergents that instantaneously denature proteins, eliminating protein denaturation concerns. The observed increased absorbance at 230 nm in DNA and RNA samples is likely due to contamination with guanidine thiocyanate. This salt is present in high concentrations in the Buffer RLT (lysis buffer) and Buffer FRN. Although additional washing steps have been incorporated to enhance the purification of the final eluate, traces of salt are still present. Nevertheless, Qiagen has shown that concentrations of up to 100 mM do not compromise the reliability of downstream applications, a finding validated during the development of this protocol. In summary, the eluates obtained through the steps outlined here should be suitable for the vast majority of molecular assays. Further optimization should only be required for assays that are sensitive to low salt contamination.

## Troubleshooting

### Problem 1

Nucleic acid yields are lower than expected.

### Potential solution

**Figure 7:**
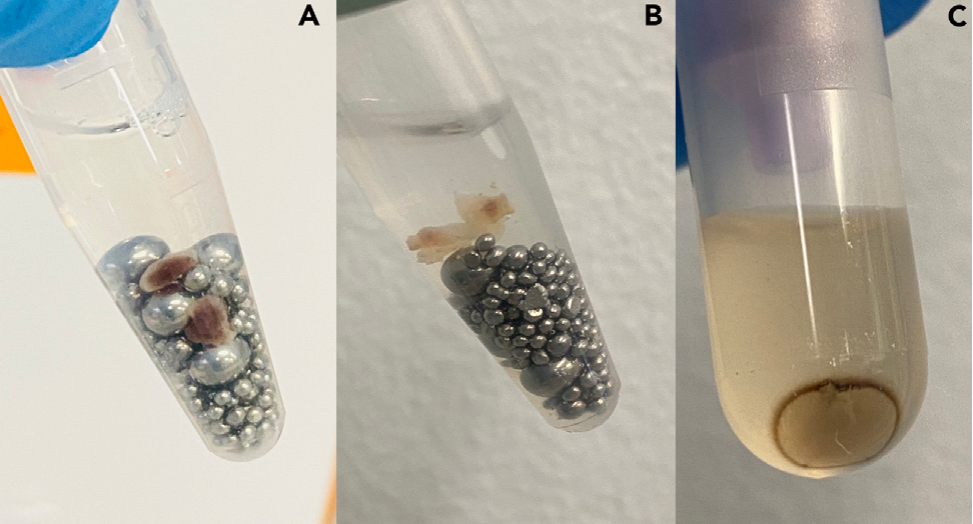
Remaining fragments and pellet after tissue disruption and sample homogenization (A) Incomplete tissue disruption. Dark tissue fragments can be observed. (B) Successful tissue disruption. The KS fragments are mainly composed of connective tissue (off-white color). (C) Pellet observed after centrifugation of the QIAShredder column.

Increase the manual homogenization time in steps 9, 14, and 15, and use the P1000 tip instead of the pestle. If larger fragments are still present after the second manual homogenization (Figure 7A), use sterile supplies to cut them into longer and thinner slices (Figure 7B) to improve disruption during mechanical homogenization.

### Problem 2

Loss of sample during disruption steps (9 and 13).

### Potential solution

Be careful not to apply excessive pressure to the frozen tissue during manual disruption. Always refreeze the tissue to preserve the loss of RNA integrity but avoid over-freezing. Over-freezing the sample creates a frozen solid tissue mass which has a greater chance of being forced out of the tube during disruption and transfer between vials.

### Problem 3

The DNA Mini Spin Column becomes partially obstructed in step 23.

### Potential solution

During the transfer of the flow-through in step 22, be careful not to disturb the pellet that may be present in the QIAShredder column collection tube (Figure 7C). The pellet contains stainless steel microparticles resulting from the collision between beads during the sample mechanical disruption. Additionally, ensure that buffers and the centrifuge temperature are within the ideal range (18°C–25°C) to avoid the formation of precipitates.

### Problem 4

The RNeasy Mini Spin Column becomes partially obstructed during centrifugation steps.

### Potential solution

Ensure that buffers and the centrifuge temperature are within the ideal range (18°C–25°C). Lower temperatures may cause precipitate formation during buffer storage and/or during processing.

## Resource availability

### Lead contact

Further information and requests for resources and reagents should be directed to and will be fulfilled by the lead contact, Larissa Scholte (larissascholte@gwu.edu).

### Technical contact

Technical questions on executing this protocol should be directed to and will be answered by the technical contact, Larissa Scholte (larissascholte@gwu.edu).

### Materials availability

This study did not generate new unique reagents.

### Data and code availability

This study did not generate/analyze datasets or codes.

## Acknowledgments

This work was supported by UM1CA181255 and UM1CA121947 from the 10.13039/100000054National Cancer Institute (NCI), 10.13039/100000002National Institutes of Health (NIH), United States. IRB-approved informed consent was obtained for all biospecimens collected.

## Author contributions

L.L.S.S.: developed, tested, wrote, reviewed, and edited the protocol; wrote and prepared figures and tables for the manuscript; is responsible for all communications among authors and with the journal; and is the primary author. J.B. tested the protocol, processed and distributed samples, and co-wrote the manuscript. D.J.N. reviewed and edited the protocol and used samples for endpoint analyses. P.S.J. tested the protocol, processed and distributed samples, and reviewed the manuscript. K.T. tested the protocol, processed and distributed samples, and reviewed the manuscript. R.S.T. tested the protocol and reviewed the manuscript. G.L. performed the statistical analyses, prepared figures, and reviewed the manuscript. S.L.L. oversaw the use of the protocol in downstream analyses and reviewed and edited the protocol. P.B. provided curated samples for protocol validation and reviewed and edited the manuscript. M.S.M. provided curated samples for protocol validation and reviewed and edited the manuscript. J.M.B. oversaw the protocol’s development, testing, validation, and sample distribution and co-wrote the manuscript.

## Declaration of interests

The authors declare no competing interests.
